# Hepatocyte growth factor and the hepatocyte growth factor receptor signalling complex as targets in cancer therapies

**DOI:** 10.3747/co.2007.108

**Published:** 2007-04

**Authors:** W.G. Jiang

**Keywords:** Hepatocyte growth factor, cMet, targeting, cancer treatment

## INTRODUCTION

Since its discovery in the late 1980s 1,2, hepatocyte growth factor (HGF) has been under intense investigation for its role in cancer. Hepatocyte growth factor has been established as a mitogen that regulates cell growth and death, a motogen that stimulates cell motility, a morphogen that modulates cell morphology and tissue or organ regeneration, and an angiogenic factor that induces angiogenesis 2,3 and, as recently reported, lymphangiogenesis 4,5.

These diverse functions of HGF and of its receptor cMet 3,6 have stimulated additional clinical interest because of their prognostic aspect and the therapeutic implications of their potential as imaging tools. Some of the recent discoveries have strongly indicated the value of both HGF and its receptor in clinical settings.

## EXPRESSION OF HGF AND ITS RECEPTOR IN CANCER

Hepatocyte growth factor—and particularly the HGF receptor—has been found to be overexpressed at the mrna and protein levels in virtually all human solid tumours, as well as in hematopoietic-derived malignancies (for a fuller list, see reference 3). Traditionally, HGF was regarded as a protein product from stromal cells in the body 2. The finding that cancer cells from epithelial origins show aberrant HGF transcripts and proteins is interesting and indicates that the source of HGF in the body is well beyond stromal. The discovery of transcription activation of *HGF* in epithelial-originated cancer cells, thought to occur through the cSrc and Stat3 pathways 7, comes in addition to the knowledge that stromal cells in tumour tissues overexpress the *HGF* transcript and HGF protein. Together with overexpressed HGF receptor on cancer cells, this situation creates a two-way stimulation for cancer cells: paracrine stimulation (HGF generated by stromal cells) and autocrine stimulation (HGF generated by cancer cells themselves). In general, paracrine stimulation is the stronger of the two in cancer, because a good number of tumour cell types are known never to express *HGF*.

The HGF receptor cMet is typically overexpressed in cancer cells and in tumour tissues in epithelial-derived tumour types, as well as in stromal and interstitial cell–derived tumours such as fibrosarcoma and other sarcoma types. An interesting finding in recent years was the discovery that, against the conventional prediction that hematopoietic-derived cells did not have the receptor, hematopoietic cells and the malignancies derived from those cells also express the HGF receptor. These sarcoma and hematopoietic malignant cells are, to some degree, dependent on *HGF* signalling for survival.

Interestingly, although the HGF receptor is a relatively large protein encoded by a large gene (the *met* proto-oncogene), genetic analyses have so far provided little information about mutation of the gene. A few reports have been published on somatic mutations, but few of those mutations are functional 8. It is therefore anticipated that aberration of the HGF receptor is largely a transcriptional event and may also occur at the post-translational level. However, some early evidence indicates that functional mutations might in fact be occurring—for example, in lymphoma cells 9. This pattern is unlikely to be a dominating one in cancer cells, and further studies are needed for clarification.

Thus, the combination of co-expression of HGF and its receptor, overexpression of the HGF receptor, and elevated levels of bioactive HGF in the tumour or circulation, or both, are frequent events in malignancies. Strong clinical evidence has showed that this overexpression or high levels of the cytokine and its receptor are intimately linked to disease progression and, in some cases, to clinical outcomes. Interestingly, HGF and its receptor, when co-expressed in the same cancer cells, predict a more virulent and aggressive tumour type 10.

## HGF AND cMet SIGNALLING, AND HGF-REGULATED GENES IN CANCER

Coupling of HGF to its receptor elicits a number of biochemical events within cells that ultimately lead to the cellular functions that are observed with HGF. These events are attributable to a number of pathways that are clearly downstream of the HGF receptor. The well-established pathways include the Rho/ Rac pathway, the phosphoinositide 3 kinase pathway, Wnt signalling, the Grb2 pathway, β-catenin–mediated pathways, and a few other pathways yet to be confirmed.

These pathways lead to the various effects of HGF on diverse cell- and tumour-type combinations. They are not unique to HGF stimulation, but they represent an impressive collection of cellular events with resulting aggressive behaviour of cancer cells, whether that behaviour be growth, migration, invasion, or other functions. Furthermore, HGF is among the most aberrant cytokines present in tumours. Thus, the HGF complex in cancer mimics, in some aspect, the classical cytokine–receptor complex abnormalities in cancer: aberrant levels of cytokine coupled with overexpressed HGF receptor and auto-activated receptor signalling pathways.

Genes that are specifically upregulated by the HGF–cMet activation were relatively less known until a recent microarray-based analysis of an *HGF-* and *met*-deleted transgenic mouse 11. A combined *HGF* and *met* gene signature with 730 regulated genes has been reported. The gene cluster covers a broad range of genes, but has a predicted pattern of genes related to cellular motility. The signature of the human homologue genes has identified a subset of liver tumours [hepatocellular carcinomas (hccs)], in which the signature has a significant correlation with an increased rate of vascular invasion and microvessel density and a decreased mean survival time of hcc patients^11^.

Other documented genes include those associated with the β-catenin pathway. Activation of cMet is able to activate the β-catenin pathway, which in turn activates transcription of the cell cycle regulators (such as cyclins). As a result, a self-amplifying cycle of unregulated growth in cancer cells is created 12–14. The cMet complex may cooperate with other signalling complexes—that is, epidermal growth factor receptor, insulin-like growth factor receptor, and neurotrophin receptor complex—in regulation of gene expression and cancer cell behaviours, including cell motility 15–17.

One of the very few genes known to downregulate the action of combined HGF and Met signalling in cells is the mitogen-inducible gene 6 (*MIG6—*also called gene 33 and receptor-associated late transducer). The *MIG6* gene can be induced by *HGF*, which in turn inhibits the intracellular activation of *met* by *HGF*. Thus, *MIG6* may be key to the self-regulation of HGF signalling in a cell. However, whether this mechanism is impaired in cancer remains to be investigated.

The other negative regulator for *met* is the *NOTCH* family. Comoglio’s group 19 has reported that the *NOTCH* receptor may downregulate *met* (a process that appears to occur at the transcription level). And *NOTCH* suppresses *HGF*-dependent Ras signalling and impairs *HGF*-dependent cellular responses. In turn, *met* activation leads to transcriptional induction of the *NOTCH* ligand Delta and the *NOTCH* effector *HES1,* indicating that *met* is able to self-tune its own protein levels and the ensuing biochemical and biologic outputs by stimulation of the *NOTCH* pathway. The Spry2 protein has also been reported to inhibit HGF-related cell proliferation, anchorage-independent cell growth, and migration in wound-healing and *in vitro* invasion assays 20. A mitochondrial protein, Mimp, that HGF is able to induce, results in growth arrest by attenuating the downstream cMet-induced events in mammary cancer cells 21.

The HGF–cMet signalling and the *HGF-*regulated genes present an opportunity in targeting and pharmaceutical manipulation. In addition, molecular complexes that inhibit cMet signalling may also provide a good opportunity for countering the action of HGF in cancer.

## HGF REGULATORS AND CANCER

The other aspect that has been considered from a targeting and therapeutic point of view is the post-translation activation of HGF. First synthesis of HGF occurs as a single chain that is biologically inactive. Activation of the inactive HGF (also known as pro-HGF) is a highly regulated process that requires orchestration of a number of enzymes. One of the most powerful activators is the HGF activator, HGFA. Other activators include urokinase-type plasminogen activator, injurin, and a number of other enzymes. A recent addition to the activator list is hepsin, which has been found to be able to cleave HGF and generate bioactive HGF 22,23. Interestingly, a number of proteins are known to regulate the activity of HGFA. Matriptase, a member of the *TMPRSS* family, is a known activator of pro-HGFA. Inhibitors of HGFA (hais) are Kunitz-domain-containing proteins that act to inhibit HGFA.

In clinical cancers, HGFA has been shown to be elevated in cancer and cancer cells. Circulating levels of HGFA are also high in patients with cancer 24. In contrast, some reports have shown that hais are present at reduced levels in cancer. However, that finding remains controversial, because in certain tumours, hais are found at a higher level. Matriptases, primarily matriptase-1, have been shown to be present at a high level in tumours 25.

In the cellular interaction between HGF and stromal cells, conditioned medium from cancer cells has recently been shown to be able to induce rapid clustering of fibroblasts and to initiate a necrotic process. The process leads to a rapid and substantial increase in the production and secretion of bioactive HGF from fibroblasts 26. The other HGFA inhibitor, hai2, has also been shown to be transcriptionally down-regulated in cancer cells, which leads to activation of Met signalling 27.

Thus, cancer involves autocrine and paracrine loops, receptor activation and mutation, gene amplification, gene rearrangement, and aberrant HGF activators and inhibitors, presenting a wide array of therapeutic targets.

## HGF AND ITS SIGNALLING COMPLEX AS THERAPEUTIC TARGETS

Given the broad functional spectrum of HGF and its receptor in cancer, targeting HGF, the HGF receptor, and signalling events has been an attractive option for cancer therapy. Therapeutic approaches have been attempted by developing tools against HGF [neutralizing antibodies, antisense oligonucleotides, ribozyme, short interfering rna (sirna), and *HGF* regulators including hais], against cMet (HGF antagonists, antibodies, small molecules, antisense, ribozymes, sirna, and non-specific inhibitors), and against cMet signalling events (coincident with anti-cMet methods), and finally by using HGF activation inhibitors. In addition, methods to mobilize the antagonistic intracellular events may also be considered. Targeting HGF and its receptor is particularly attractive in cancer therapies, because HGF is a dual player in the complex biology of cancer development and progression: it acts directly on and stimulates cancer cells, and it acts as an angiogenic factor and lymphangiogenic factor that aid the growth and spread of cancer cells.

These therapeutic approaches are largely in the development phase, with a small number—mostly non-specific inhibitors to cMet—now in early clinical study. The list of these developments is beyond the scope of the current article. However, some of the early developments, such as the HGF antagonist and small molecules, are anticipated to have an opportunity to make it into human-phase studies.

## HGF AND ITS RECEPTOR AS IMAGING TARGETS

The fact that the HGF receptor is highly overexpressed in cancer cells, and indeed well-expressed in endothelial cells, has prompted interest in exploring the receptor as potential tool for imaging. Conjugated anti-cMet antibody has been reported to be able to light up small tumours *in vivo.* If that result proves valid in clinical settings, then a wider implication arises: by conjugating therapeutic agents to the antibody, the technology might be used in diagnostic imaging and in therapies. The conjugates will serve as a delivery vehicle and at the same time as a receptor-neutralizing agent (for a neutralizing antibody).

Using ^125^I-labelled anti-Met antibody in *in vivo* models, the antibody has been shown to be enriched in the *met-*positive tumour of lung cancer (SK-LMS-1 xenograft) 28, suggesting a possible diagnostic and therapeutic advantage. Vande Woude’s group 29 has developed a range of anti-Met antibodies for *in vivo* imaging. In their recent report, tumours were shown to exhibit rapid and sustained uptake of these ^125^I-labelled antibodies, permitting detection of *in vivo* tumours by a total-body gamma camera 29. Infusion of HGF increases blood flow and oxygenation in organs that have high levels of the HGF receptor and in smaller vessels in tumours, suggesting a potential use of functional molecular imaging in cancer 30.

One interesting challenge of using anti-cMet antibody would be the presence of soluble cMet proteins in the circulation because of shedding from cancer cells. Ectodomain shedding of the HGF receptor has been recently reported 31. Shed receptor can be detected in tumour-bearing plasma and in cell culture supernatant, and is linked to tumour progression. Shedding may reflect the increased rate of receptor synthesis or of activation-enzyme activity in cancer cells (or both), resulting in degradation of the receptor. The presence of this circulating protein may therefore impede the action and effectiveness of antibodies raised against the extracellular domain of the receptor.

## PERSPECTIVES

It is compelling that HGF and its receptor are key players in the development and, importantly, in the progression of solid and blood-borne malignancies. The cytokine and its receptor complex have significant prognostic value in cancer. But despite all these optimistic biologic and clinical aspects, it must be borne in mind that HGF is one of the numerous protein factors associated with cancer and cancer progression. The unique niche for HGF is its double role in cancer: direct action on cancer cells to increase their aggressiveness, and direct action on endothelial cells to induce angiogenesis and lymphangiogenesis. This role makes HGF–cMet complex a highly desirable target. Thus, beyond the complex’s traditional prognostic and predictive value is the apparent strong implication that HGF–cMet would be a promising molecular target and molecular imaging tool.
